# Smart Feeding Unit for Measuring the Pecking Force in Farmed Broilers

**DOI:** 10.3390/ani11030864

**Published:** 2021-03-18

**Authors:** Rogério Torres Seber, Daniella Jorge de Moura, Nilsa Duarte da Silva Lima, Irenilza de Alencar Nääs

**Affiliations:** 1 School of Agricultural Engineering, University of Campinas, Campinas, Av. Cândido Rondon, 501 Barão Geraldo, São Paulo 13083-875, Brazil; rogerio.torres.seber@gmail.com (R.T.S.); djmoura@unicamp.br (D.J.d.M.); 2 Graduate Program in Production Engineering, Paulista University, São Paulo 04026-002, Brazil; nilsa.lima@stricto.unip.br

**Keywords:** broiler, feeding system, pecking force, precision livestock farming

## Abstract

**Simple Summary:**

We present a novel method for assessing broiler pecking force data during feeding. The prototype consisted of a power supply unit with a data acquisition module, management software connected to a computer for data storage, and a video camera to verify the pecking force during signal processing. The acquisition, processing, and classification of the pecking force signal information were valuable during broilers’ feeding. The smart feeding unit (SFU) prototype was useful in the continuous generation of information that could be applied to evaluate the amount of pecking force and performance during the broilers’ growth.

**Abstract:**

Feeding is one of the most critical processes in the broiler production cycle. A feeder can collect data of force signals and continuously transform it into information about birds’ feed intake and quickly permit more agile and more precise decision-making concerning the broiler farm’s production process. A smart feeding unit (SFU) prototype was developed to evaluate the broiler pecking force and average feed intake per pecking (g/min). The prototype consisted of a power supply unit with a data acquisition module, management software connected to a computer for data storage, and a video camera to verify the pecking force during signal processing. In the present study, seven male Cobb-500 broilers were raised in an experimental chamber to test and commission the prototype. The prototype consisted of a feeding unit (feeder) with a data acquisition module (amplifier), with real-time integration for testing and intuitive operation with Catman Easy software connected to a computer to obtain and store data from signals. The sampling of average feed intake per pecking per broiler (g) was conducted during the first minute of feeding, subtracting the amount of feed provided per the amount of feed consumed, including the count of pecking in the first minute of feeding. An equation was used for estimating the average feed intake per pecking per broiler (g). The results showed that the average broiler pecking force was 1.39 N, with a minimum value of 0.04 N and a maximum value of 7.29 N. The average feed intake per pecking (FIP) was 0.13 g, with an average of 173 peckings per minute. The acquisition, processing, and classification of signals in the pecking force information were valuable during broilers’ feeding. The smart feeding unit prototype for broilers was efficient in the continuous assessment of feed intake and can generate information for estimating broiler performance.

## 1. Introduction

Broiler production contributes significantly to Brazilian agribusiness. In 2019, Brazilian broiler meat production volume was 13,245 metric tons, corresponding to 13.4% of the world market (98,594 metric tons). The poultry industry is evolving to meet the global demand for animal protein with low environmental impact [1,2,3], with integrated, vertical production, and applying technologies in the production process. Such actions aim to increase the productive efficiency index [4,5], improve the welfare and health of animals [6,7] with lower production costs without compromising parameters of welfare, performance, and quality [8]. These initiatives also lead to improving the consumer perception of broiler meat [9]. The use of technology is essential to manage modern broiler farms, and providing relevant information to farmers enhances their decision-making during the production cycle.

Intelligent equipment with a rapid response in real-time has been extensively studied and used in the poultry production process. Besides contributing to precision livestock farm development, these studies apply connectivity and analysis tools in real-time and use algorithms to monitor the production cycle during animal growth, behavior, welfare, health aspects, and performance [10,11,12,13,14,15,16,17,18]. Current literature presents developed equipment for poultry farming using sounds, images, and modeling of signals of force and pecking sounds to monitor animals’ growth and other factors related to the welfare, behavior, and feeding of birds [11,16,19,20,21,22,23]. Although the use of an automatic recording of sounds for animal husbandry and health management (quantitative analysis) in other species, such as swine, show similar results in automatic and manual assessments of the frequency of coughing, the disadvantage of manual assessments is the time spent compared to that of automatic assessments [24].

Animal feeding is one of the essential processes during broiler production and one of the most studied subjects with methods of analyzing sound signals and video images. Previous studies aimed to assess broilers’ food consumption using scales, including pecking sounds and developed a pecking classification algorithm for continuous and non-invasive broiler production monitoring [21,22]. Integrating the previously studied variables, a pecking detection system including video footage, a microphone to record sound signals, a scale to record bird weight automatically, and the use of a group pecking classification algorithm were used to evaluate the short-term broiler feeding behavior [11]. An automated system (group-housed individual turkey feeder and bodyweight measurement station) was developed to monitor the turkeys’ feeding and body weight in real-time. The monitoring system consisted of hardware and software subsystems (hardware subsystem: mechanical framework of feed stations, radio frequency identification components, electronic scales, communication modules, and a central computer; software subsystem: a hardware monitoring and data acquisition program, and a data processing and management program). The system was tested with a group of turkeys to assess data on the frequency of feeding behavior and performance [25]. Another study using signal analysis methods developed a chicken pecking force equation on an automatic feeder. The equation involves mathematical and statistical approaches to analyze the chicken pecking force at different stages of production. The pecking force was related to parameters of feed flow rate and more accurate decision-making regarding the hopper aperture in the feeder at the production process [23]. The results indicated that the birds’ satiety level determined the pecking force described by a polynomial function [23].

The coupled cranial kinesis (the ability to move the upper beak relative to the braincase [26]) in domestic fowl does not play a dominant role in the feeding process. The jaw drops just after lifting the upper jaw, suggesting that the coupled cranial kinesis does not necessarily happen while the food is grasped [16]. Similar traits may occur in the following cycles for the food going into the oral cavity during the feeding process. However, the coupled kinesis is applied when the bird closes the beak since it cannot depress the upper jaw without raising the lower jaw [27]. However, the bird can adapt specific beak movement depending on the type of food, and such behaviors are subordinate to the constraints of the beak morphological structure [16]. Foraging is a natural bird behavior, and during foraging, it also pecks the ground, and often broilers pecking does not result in the retention of a feed particle [28]. Therefore, we need to continuously check the head and beak movements to assess feed behavior and consequent performance. 

In the current literature, we did not find a study that has directly measured the signs of broiler head movement to identify and classify the pecking force during feeding that was specially instrumented for monitoring broiler feeding behavior. We believe that in the future, such equipment associated with the signal interpretation may provide us with a unique ability to manage production data regarding feed performance and detection of numbers of birds per feeder in a non-intrusive way. An intelligent feeding unit can collect data from force signals and transform it into information such as the pecking force during the feed intake. Such continuous information such as feed intake per pecking, activity, number of birds around the feeder, and weight gain by feed intake allows faster and more accurate decision-making regarding the farm level’s production process. Therefore, the present study aimed to develop a prototype of an intelligent feeding unit (smart feeding unit, SFU) to evaluate and register the broilers’ pecking force during feeding by correlating the force applied with the actual catch of feed particles.

## 2. Materials and Methods 

The development of the SFU consisted of constructing a prototype and commissioning the prototype for the acquisition of broilers’ pecking force data.

### 2.1. The Prototype of the Smart Feeding Unit

**Figure 1:**
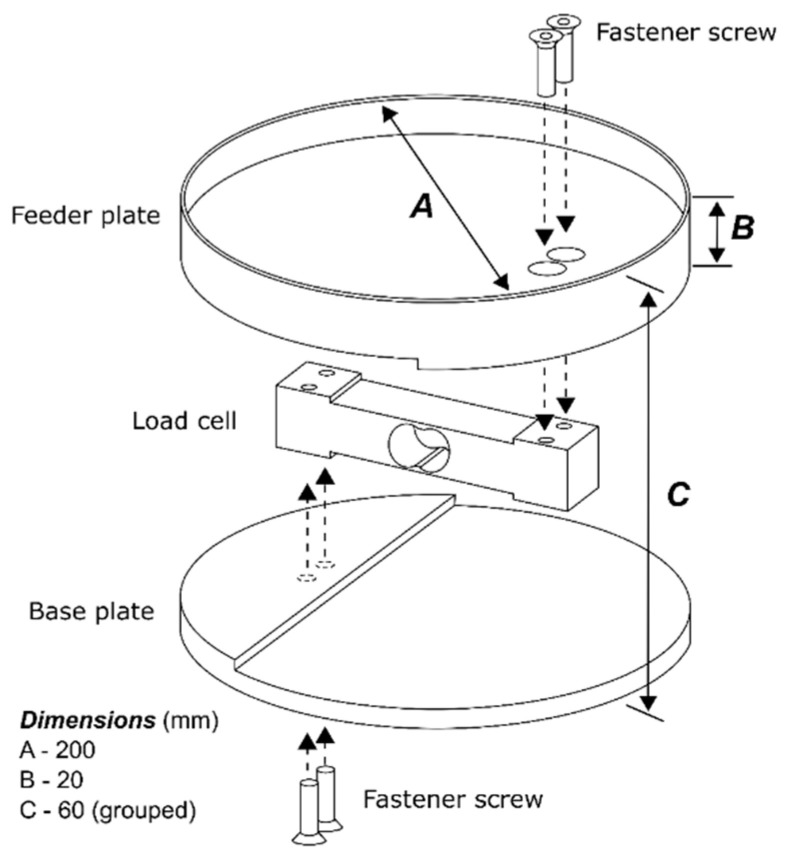
Schematic of the smart feeding unit prototype structure.

Figure 1 presents the structure of the feeding unit. The parts that bring up the SFU are a 200 mm diameter feeder plate with a 20 mm height, a load cell (manufacturer Hottinger Baldwin Messtechnik—HBM), a base plate, and a fastener screw. The prototype was built using modular steel components. The feeding unit’s height was adjusted according to the bird’s height (as it grows). The equipment was installed on concrete support to maintain proper stability.

The prototype was subjected to tests during the adaptation phase of the birds to the new feeder. The prototype test started in the last week of the production cycle (35–41 days old), and validation was performed on the last day of the production cycle, lasting 24 h to collect signals from the birds’ pecking force.

### 2.2. Experiment

Seven male Cobb-500 broilers were reared from 1 to 42 days old, considering the first five weeks (1 to 35 days old) as an adaptation phase to the environment, and in the last week, the test of the feed unit prototype (35 to 42 days old). From 35 days of age on, an instrumented feeder was made available to measure the pecking force during feeding (through a week before slaughter). Seven broilers were housed in an experimental floor chamber (experimental environmental controlled chamber) equipped with a tubular feeder (for the adaptation phase of the broiler feed), a pendant drinker, temperature sensors, air humidity control, electrical heater (for the initial rearing phase), air exhaust fan, mechanical cooling, and dimmable LED lighting (artificial light). These devices make the flow of heat supply and removal, vapor supply and removal, illumination, environmental sensing and control, and video monitoring.

The floor was covered with 5 cm thick wood shavings litter, which was reloaded whenever necessary. The daily lighting was 16 h during the growth period. Feeding and water were provided ad libitum during the experiment.

The experiment was carried out at the Animal Environmental Laboratory at the School of Agricultural Engineering at the University of Campinas (Unicamp, Brazil), and the study was approved by the University’s Animal Ethics Committee (protocol number 5278-1/2019—CEUA—Unicamp).

### 2.3. Data Acquisition and Signal Processing

**Figure 2:**
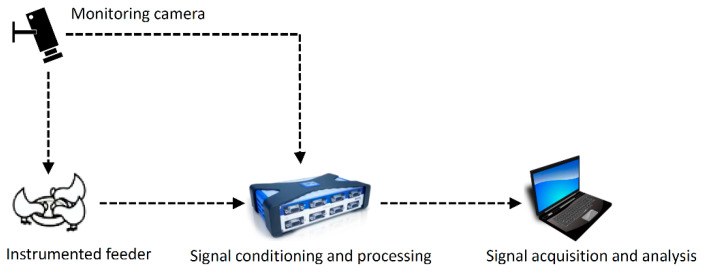
General scheme of signal conditioning and processing for data acquisition.

The data acquisition and signal processing consisted of the data acquisition module (QuantumX—MX840A amplifier, manufacturer Hottinger Baldwin Messtechnik—HBM), with real-time integration for testing and Catman Easy software (CatmanEasy version 4.2, manufacturer Hottinger Baldwin Messtechnik—HBM), connected to a computer for obtaining and storing data from signals (Figure 2). Data acquisition is the process of obtaining via sampling a signal from a sensor and converting it to an electrical value (usually a voltage level) and later conversion to a digital value for further computer processing. Moreover, sensors are the devices that convert one type of electrical or mechanical signal (input-signal) into another (output-signal), usually an electrical signal. Signal conditioning is a step of data acquisition that combines the signal emitted by the sensor installed in the feeder (input-signal), amplifying, filtering the noise, and converting an analog signal into a digital signal (output-signal), with the input in a computer. The signal amplification is the increasing signal for processing (or digitization) that can increase the signal input resolution or increase the signal-to-noise ratio. In signal conditioning, the frequency spectrum is filtered only to include the valid data and block any noise [29]. A video camera (Sharp Corporation, 470 lines with 3.6 mm converging lens) was utilized for acquiring the images for checking of pecking during signal processing for data analysis (synchronizing images and signals), maintained in continuous monitoring mode (Figure 2). The video images were synchronized with the acquisition of signals to validate when birds pecked to determine the average feed consumption per pecking.

The software used to acquire, visualize, analyze, and report signal measurement data was Catman Easy (CatmanEasy version 4.2, manufacturer Hottinger Baldwin Messtechnik—HBM) to ensure the synchronism between the feed pecking image and the force signal acquisition of the pecked feeder. The signal analysis aims to extract information from the data acquired to generate some desirable information [30]. The feed pecking signals data reports during all the broilers feeding were registered and sent automatically to a computer. It was also possible to visualize the signal using graphical output.

### 2.4. Classification of Pecking and Validation

The automated real-time smart feeding unit prototype was tested in the last week of the broiler rearing cycle for system validation. The data collected by the data acquisition system contained raw data (with noises generated during the birds’ movement and feeding) that were processed, organized, and filtered to remove the noise and later classify the signals of feed pecking and other eventual beak movements in the feeder. Video analysis was used as a tool in the validation of feed pecking (pecking vs. other beak movements). Image and signal force were synchronized for the pecking validation, ensuring that the feed was eaten. All force sampling occurred synchronically with the head-movement image acquisition, confirming that the registered pecking force originated from an observed feeding. The sampling selected was 1 min during bird feeding (sample size *n* = 284). Feed intake was estimated during a sampling synchronized image and signal force. The feed intake was calculated by subtracting the amount of feed provided (excluding the feeder’s weight) per the amount of feed consumed, including the count of pecking in the one-minute concerning feeding intake. 

Feed ration consumption was automatically assessed by the sensor system installed in the feeder and processed using the data acquisition system installed in the computer (Figure 2). The average feed intake per pecking per broiler (g) was estimated using Equation (1), adapted from Aydin et al. [21].

(1) $$FIP\;\left(g\right)=\;\frac{TFI\left(g\right)}{TNP},$$

where *FIP* is feed intake per pecking, *TFI* is total feed intake (g), and *TNP* is total number of peckings per minute (*TNP*).

### 2.5. Statistical Analysis

The automated real-time smart feeding unit prototype was tested in the last week of the broiler rearing cycle for validation. The data collected by the data acquisition system contained raw data (including noises generated during the birds’ movement and feeding) that were processed, organized, and filtered to remove this noise and later classify the signals (pecking vs. non-pecking). The comparison of the accurate feed pecking patterns and other noises were classified to define feeding behavior. 

A table was generated from the data acquisition software containing the filtered and standardized data for the descriptive analysis and the *t*-test for a sample (one-way *t*-test) [31,32]. A one-way test is a hypothesis test that counts the chance of results only in one direction [31,33].

The descriptive analysis and *t*-test were applied to a sample size of 284 pecking force, measured in Newton (N). The average pecking force (6.5 N) of young chickens (<8 weeks old) tested in a poultry feeder (smaller hopper aperture) was utilized for the alternative hypothesis in the *t*-test [23]. The hypothesis test was presented, H0: all means are equal, vs. H1: at least one mean is different [32]. The data were analyzed using PAST software [34] (Paleontological Statistics version 4.03).

Sample tests are used to determine whether a single sample comes from a population with a given hypothetical average (alternative hypothesis µ_0_). The alternative hypothesis is the data from the birds’ pecking force (single sample) are equal to the mean of pecking force found in a previous study [23]. For the *t*-test of a sample (parametric), the confidence interval was 95% for the difference in means based on the standard error for estimating the mean and t distribution. The *t*-test statistic was calculated as expressed in Equation (2) [31]:

(2) $$t=\;\frac{\bar{x}\;-\;{µ}_{0}}{\frac{S}{\sqrt{n}}}$$

where $\mu_{0}$ is the hypothesized population mean, $\bar{x}$ is the sample mean, *n* is the sample size (number of observations), and S = is the sample standard deviation. Under the null hypothesis, the test statistic has Student’s *t* distribution with *n* − 1 degree of freedom.

## 3. Results

**Figure 3:**
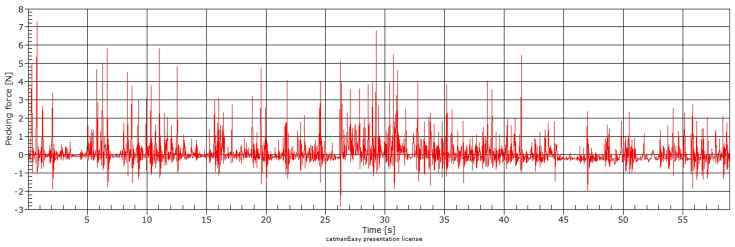
Pecking force at one-minute time length.

**Figure 4:**
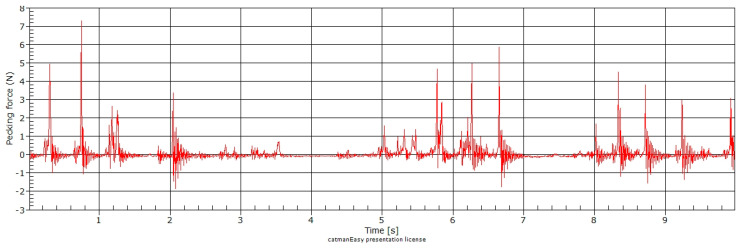
Pecking force at ten-second time length.

The test of the smart feeding unit (SFU) prototype allowed the acquisition of the pecking force data instantly during the broiler feeding. Figure 3 shows the pecking force data acquisition in one minute during the broilers’ feeding, and Figure 4 shows the pecking force in ten seconds. The intensity and speed of data collection are due to the equipment’s sensitivity in detecting the force when the broiler feeds.

**Figure 5:**
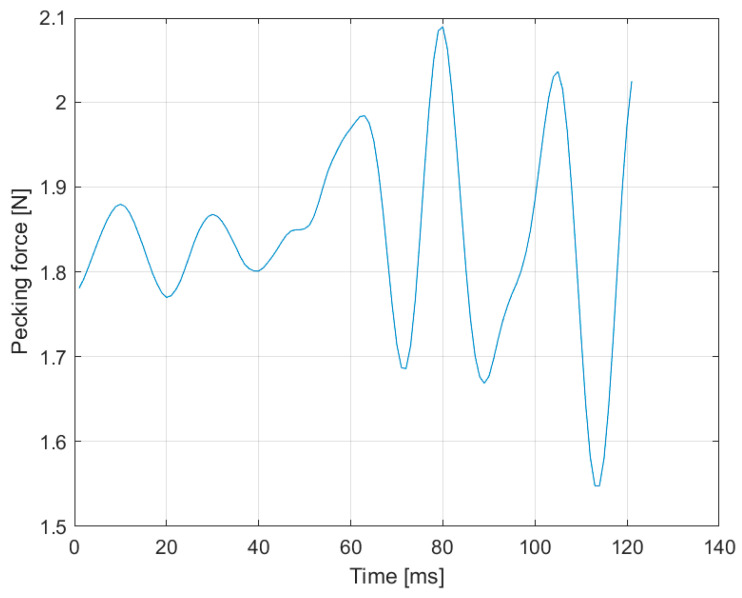
Pecking force at 120 ms.

**Figure 6:**
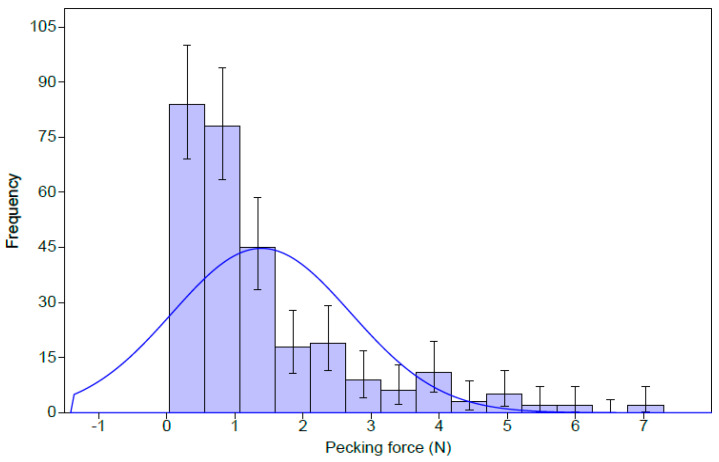
Frequency histogram of pecking force values.

We observed a pecking force pattern during broiler feeding in the three images (Figure 3, Figure 4 and Figure 5). Figure 6 represents the pecking force in 120 ms (milliseconds) described as one peck, showing the force variation applied to the feeder’s sensor. The average feed intake per pecking (*FIP*) was 0.13 g, with an average of 173 peckings per minute (pecking frequency).

The frequency distribution (histogram) with a normal fit adjusted in the histogram (Figure 6) shows a tilt of the tail to the right due to the birds’ peak pecking force. 

The results of descriptive statistics (Table 1) show that the average broiler pecking force was 1.39 N, with a minimum value of 0.04 N and a maximum value of 7.29 N from the analysis of 284 samples. The results of the *t*-test indicate that the mean (1.39 ± 0.15 N) of the broilers’ pecking force differs (*p* < 0.001) from the average birds’ pecking force in general (Table 1).

The classification of force signals in pecking was performed efficiently with the aid of synchronized video images to identify an effective pecking in a group or individual. 

## 4. Discussion

The results showed that the broilers’ average pecking force was validated by the smart feeding unit (SFU) prototype designed for fast response, with efficient readings for measuring force signals. We also observed a cyclic pattern of pecking force during broilers feeding that can be explained by the phases of the food intake process, specifically related to the biomechanical movement of the broiler’s head [16,35]. A previous study evaluated broilers’ biomechanical movement during feeding through computational analysis of images classifying the sequences of frames and kinematic variables to analyze the biomechanical behavior [35]. The authors considered six mandibulations (two sequences of three mandibulations) that involve a cycle of beak opening and a beak closing cycle. The studied kinematic variables included the head’s displacement, the speed, and acceleration of beak opening and closing. Their results indicated that the birds’ feeding behavior is divided into two phases, an appetite phase (exploration in search of feed) and a phase of actual feed consumption. Another relevant result was that birds are selective about the food’s particle size in the initial phase of the production process. The authors concluded that birds’ biomechanical patterns are related to different types of feed [16].

Another study carried out with hens of different ages (<8 weeks, 8–5 weeks, and >52 weeks old—weeks old birds), using an automatic feeder with different openings (hopper aperture: smaller, intermediate, and larger), developed a pecking force equation that was related to the feeding and hopper aperture of the feeder. The results showed that the birds’ satiety level determined the pecking force. The maximum pecking force of chickens younger than eight weeks of age was 10 N after 40 min of feeding for the largest hopper opening. The pecking force was lower in the smaller feeder. The amount of feed consumed also decreased with the feeding time, indicating the birds’ satiety leads to a lower pecking force [23]. The maximum broiler pecking force found was 7.29 N. Considering there are differences in feeder type, and the subject was broiler and not chicken, the average force for chickens, regardless of age, differed from those of the present study.

Other studies have also analyzed methods for assessing the broiler feed process [11,21,22]. However, the authors used sound signals as the primary method of detecting the broiler pecking. Pecking sounds from 10 broilers of 39-day-old were assessed while feeding, associated with video image records. The feeding system also registered the birds’ weight simultaneously, and from there, they developed an algorithm to detect the birds pecking in groups. The feed intake was estimated automatically after the classification of the individual pecking sound that was detected by the algorithm. However, the proposed system did not exceed 90% accuracy in detecting broilers’ pecking sounds when they were in group feeding because of the overlapping pecking sounds. The correlation was high considering the feed intake from broilers’ pecking sound analysis. The authors concluded that a non-invasive and automated continuous system for monitoring chickens’ feeding behavior could be essential for monitoring the growth process [22]. A previous study by the same group developed an algorithm for detecting broiler pecking to classify individual pecking and monitor feed intake. The pecking was accurately classified (93%), and feed consumption was correctly monitored (90%) from the sound analysis [21]. When analyzing the broilers’ feeding behavior in the short term at a group level, including a system of evaluation by sound, image, and weighing monitored in real-time, the authors found a positive correlation between the used methods. The estimated precision was 90% when analyzing the meal size, 95% when evaluating the meal duration, 94% when studying the number of meals per day, and 89% for the feeding rate of broilers at 39 days of age from the analysis of the pecking sound and the bird weighing in an instrumented feeder [11].

In turkey production, an automated feed consumption and body weight monitoring system was also evaluated to assess feeding behavior. The automated system was based on ethernet and multithreading programming for real-time data acquisition. The authors [25] concluded that the system was effective in the acquisition and management of raw data and in the extraction of information on feeding behavior, which included the distribution of feeds over time, feed conversion rate at different stages of growth, pecking force, deglutition intervals, and meal breaks during feeding during turkey rearing.

Aydin et al. [21], using sound analysis, found that the 28-day-old broiler average feed intake per pecking was 0.025 g, and the average number of peckings was 85/min per broiler. However, the present study was conducted at 42 days of age and different strains. The average feed intake per pecking was 0.13 g, and the average number of peckings was 173/min per broiler. These results indicate that the equipment was fit to monitor broiler feeding. Broilers’ age might explain the higher feed intake and the larger amount of pecking since intake increases according to the growth stage [36]. Further studies are needed to assess the relationship between feed intake and pecking force during feeding and at all stages of rearing.

The integration of smart sensors and technologies in the broiler rearing processor helps producers to optimize and minimize production losses and improves monitoring of birds in real-time [37,38,39]. The present study provided valuable information about an automatic broiler feeding system that collaborates with the application and implementation of intelligent systems as a data management tool during the broiler production process, potentially contributing to precision livestock farming to monitor the welfare of birds.

## 5. Conclusions

The smart feeding unit (SFU) as a broiler feeder has been tested and validated for its application to measure the bird’s pecking force. The acquisition, processing, and classification of signals in information on the pecking force were valuable during broilers’ feeding. The results confirmed that the average broiler pecking force was 1.39 ± 0.15 N and the average feed intake per pecking was 0.13 g, with an average of 173 peckings per minute.

This equipment can generate information that can serve as a basis for further studies on the performance, behavior, welfare of broilers, and automation of rearing processes regarding broiler feed management and can be easily adapted and included in other systems already on the farm.

## Figures and Tables

**Table 1:** Summary of results of descriptive analysis and *t*-test of broilers’ pecking force.

Statistical Summary			Lower Confidence Limit	Upper Confidence Limit	
*n*		284	284	284	
Minimum		0.04	-	-	
Maximum		7.29	-	-	
Mean		1.39	1.24	1.54	
Standard error		0.08	0.07	0.09	
Standard deviation		1.31	1.15	1.49	
**One-sample *t*-test—Pecking force**					
*t* Statistic	*p*-value (2-tailed)	df ^1^	Mean difference	95% Confidence interval of the difference	
				Lower	Upper
−65.66	<0.0001	283	5.11	1.24	1.54

^1^ df: the degrees of freedom for the test, df = *n* − 1.

## Data Availability

Data will be available upon request to the first author.
